# Deciphering the Role of Regulatory CD4 T Cells in Oral and Oropharyngeal Cancer: A Systematic Review

**DOI:** 10.3389/fonc.2018.00442

**Published:** 2018-10-15

**Authors:** Caoimhín O'Higgins, Frank J. Ward, Rasha Abu Eid

**Affiliations:** ^1^Institute of Dentistry, School of Medicine, Medical Sciences and Nutrition, University of Aberdeen, Aberdeen, Scotland; ^2^Institute of Medical Sciences, School of Medicine, Medical Sciences and Nutrition, University of Aberdeen, Aberdeen, Scotland

**Keywords:** regulatory T cells, oral cancer, oropharyngeal cancer, patient outcome, tumor microenvironment

## Abstract

**Background:** Recruiting regulatory CD4 T cells (Tregs) into the tumor microenvironment is an important tumor escape mechanism. Diminishing these suppressive cells is therefore one of the targets of cancer immunotherapy. Selective depletion of Tregs has proven successful in enhancing anti-tumor immunity and therapeutic efficacy in multiple tumor types. However, the role of Tregs in oral/oropharyngeal cancers is unclear with conflicting evidence regarding the effect of these suppressive cells on tumor prognosis. In this study, we sought to review the role of Tregs in oral/oropharyngeal cancer with the aim of deciphering the controversy regarding their effect on tumor progression and prognosis.

**Methods:** A systematic review of the literature pertaining to the role of Tregs in oral/oropharyngeal cancer was performed using Scopus, Embase, and PubMed. Forty-five records were deemed eligible and data describing methodology of Treg detection, tumor type, and association with prognosis were extracted.

**Results:** Of the 45 eligible manuscripts accepted for this systematic review, thirty-nine studies reported data from human subjects while the remaining studies focused on animal models. Sixteen studies were carried out using peripheral blood samples, while samples from the tumor site were analyzed in 18 studies and 11 studies assessed both blood and tumor samples. The transcriptional factor, Foxp3, was the most commonly used marker for Treg identification (38/45). The findings of 25 studies suggested that an increase in Tregs in the tumor microenvironment and/or peripheral blood was associated with poorer prognosis. These conclusions were attributed to the suppression of immune responses and the consequent tumor progression. Conversely, nine studies showed an increase in Tregs in peripheral blood and/or tumor microenvironment was related to a favorable prognosis, particularly in the presence of human papilloma virus (HPV), the status of which was only assessed in 11 studies.

**Conclusions:** This review underlines the importance of host immunity in the behavior of oral/oropharyngeal cancer. Furthermore, we report an apparent lack of clarity regarding the true role Tregs play in oral/oropharyngeal cancer progression which could be attributed to inconsistent detection techniques of Tregs. Our results therefore highlight the need for clearer methodologies and more robust phenotyping when defining Tregs.

## Introduction

Head and neck cancer is the sixth most common malignancy with an estimated 686,000 new cases and 375,000 deaths reported annually (combined worldwide laryngeal, oral, and pharyngeal cancer incidence) (1). The majority of head and neck cancers are squamous cell carcinoma (SCC). Along with alcohol consumption, smoking and various forms of betel quid chewing [which have long been associated with the development of oral and oropharyngeal squamous cell carcinoma (OPSCC)], it is now recognized that human papilloma virus (HPV) infection plays an important role in the onset of HPV positive OPSCC (2).

Despite advances in diagnosis and treatment, OPSCC mortality rate has improved little over the years, with 5 year survival rates as low as 53% reported in England for cancers of the oral cavity (3). This is mainly attributed to late diagnosis and the absence of predictors of disease progression in oral premalignant lesions.

Recently a growing emphasis is being placed on the role of the immune system and its association with the occurrence and progression of cancer. Indeed, cancer immunotherapy is among the most important developments in cancer treatment. It was therefore not surprising that cancer immunotherapy was named the scientific breakthrough of the year in 2013 (4). Despite the impressive successes in cancer immunotherapy, the response in patients is sometimes short lived. This is due to factors that hamper the immune response against cancer such as the presence of the suppressive regulatory CD4 T cells (Tregs) in the tumor microenvironment (5).

Tregs are a subpopulation of CD4^+^ T lymphocytes which are capable of discerning self-antigens from non-self-antigens and suppressing the expansion of effector cells directed against self. The major subpopulations of Tregs include thymus-derived Tregs (tTregs), Tregs which have been induced peripherally by different cytokines (pTregs), and induced Tregs which are induced *in vitro* in the lab, (iTregs). All Treg types maintain regulatory functions, and their development and function are thought to be dependent on the expression of the transcription factor Forkhead box P3 (FoxP3), known as the “master regulator” of Treg regulatory functions (6, 7).

Within the tumor microenvironment, Tregs have an opposing action to cytotoxic CD8 T cells (8), and reducing the number of Tregs was found to reinvigorate anti-tumor immunity and promote tumor regression in different types of cancer (9–14).

The role of Tregs in oral/oropharyngeal cancer is not fully understood and different studies have reported conflicting evidence regarding the role of Tregs in oral/oropharyngeal cancer progression and prognosis. Some studies emphasized the suppressive role of Tregs within the tumor microenvironment or the periphery, thus negatively impacting the patient clinical outcome (15–20), others reported a positive clinical outcome associated with an increase in circulating or tumor infiltrating Treg (21–29).

It is therefore important to fully comprehend the causes of these contradictions to enable full understanding of the role that Tregs play in oral/oropharyngeal cancer. This will enable designing novel immune-therapeutics that optimize the anti-tumor immune response and ultimately clinical outcome.

In this study, we sought to review the role of Tregs in oral/oropharyngeal cancer with the aim of deciphering the controversy regarding their effect on tumor progression and patient prognosis.

## Methods

We conducted and reported this systematic review following the PRISMA statement (30).

### Search strategy

A systematic search of PubMed, Embase, and Scopus (from their commencements to May 2017 when the search was performed), for studies in the English language with no species restrictions and for studies related to the role of Tregs in oral and oropharyngeal cancer. The following keywords were used in searching: (“head and neck cancer” or “head and neck malignancy” or “oropharyngeal”) and (“epithelial dysplasia” or “oropharyngeal premalignancy”) and (“tumor microenvironment” or “cancer immunology” or “tumor infiltrating lymphocytes” or “TILs” or “circulating immune cells” or “peripheral immune cells”) and (“regulatory T cells” or “regulatory T lymphocyte” or “regulatory CD4 T lymphocyte” or “regulatory CD4 T cells” or “Treg” or “Tregs” or “Foxp3+ or CD4+Foxp3+” or “CD25+” or “CD4+CD25+” or “suppressive immune cells” or “suppressive lymphocytes”).

We scrutinized the reference lists of the identified reports, reviews, meta-analyses, and other relevant publications to find additional pertinent studies. The “related articles” function was also used to broaden the search.

Our inclusion criteria were:

1. Studies must have been published as original articles
2. Studies must have been published in English
3. Studies assessing the role of Tregs in oral and oropharyngeal cancer.

Our exclusion criteria were:

1. Letters to the editor, conference abstracts, review, and systemic review articles
2. Studies that focused on thyroid, laryngeal, esophageal, and salivary gland tumors.

### Data extraction

The studies which met the inclusion criteria were summarized and data extraction was performed using a pre-defined form by one of the authors (CO) and accuracy checks were performed on over 75% of the manuscripts by (RA). Data extracted included: author, journal, year of publication, sample size, tumor type, tumor site, species, whether blood or tumor sample were used, method of sample analysis, markers used to detect Tregs, role of Tregs in tumor progression/prognosis, HPV status, correlations between HPV status and Tregs, tTregs vs. pTregs, and any data related to oral epithelial dysplasia.

Due to the huge variation in the study designs, the number of samples, the tumor site, and the method for detecting Tregs within tumor or blood samples, meta analyses of the results were not possible.

## Results

### Manuscripts included in the systematic review

**Figure 1:**
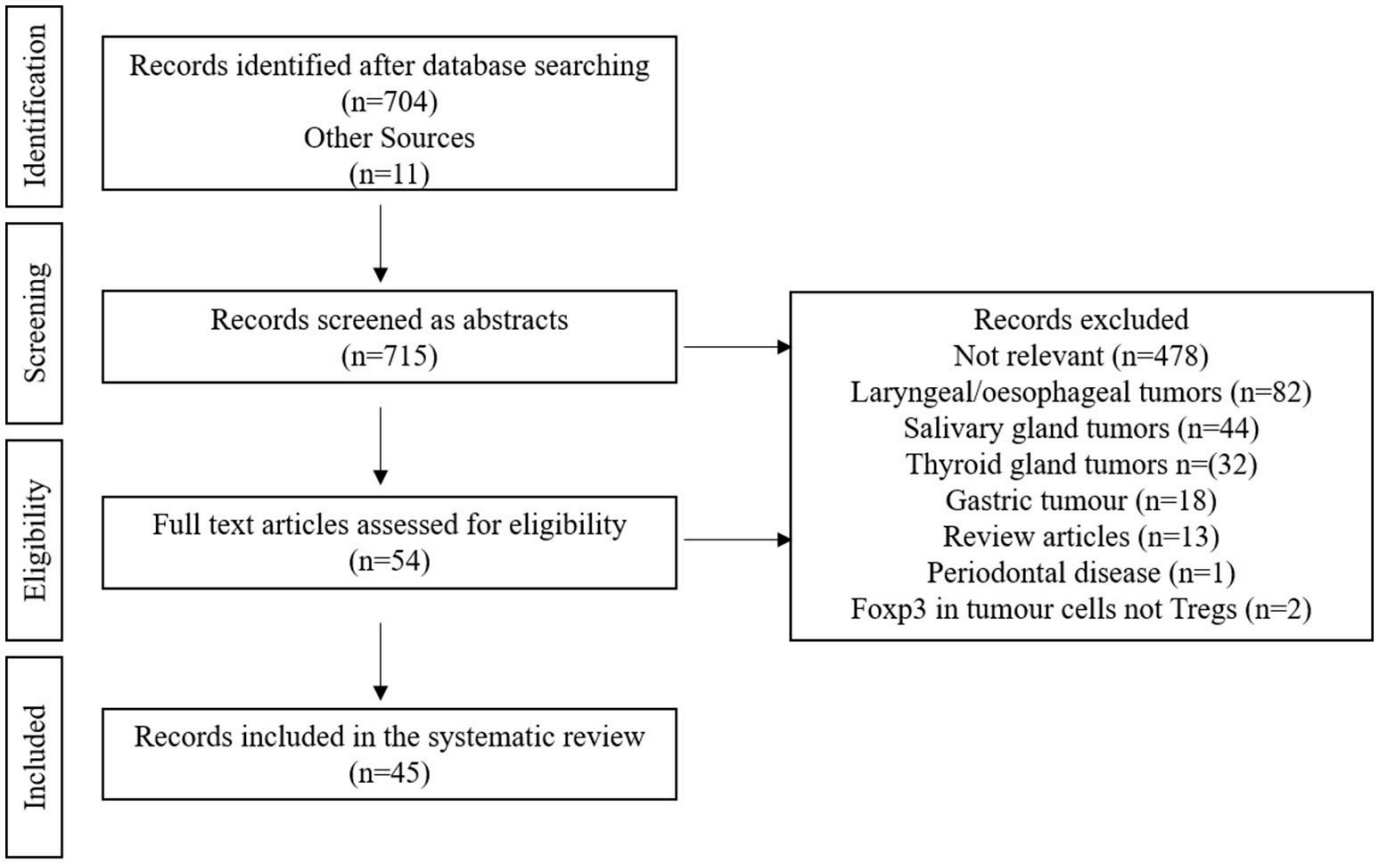
Flow diagram of the studies retrieved for the review.

Of 715 identified citations, we identified 54 articles which met the inclusion criteria. Following full text screening, 45 articles were deemed to be eligible for inclusion in this study. Reasons for exclusion included irrelevant manuscripts which did not tackle the role of Tregs in oral and oropharyngeal cancer (*n* = 478), manuscripts that focused on tumors other than oral or oropharyngeal; laryngeal/esophageal (*n* = 82), salivary gland (*n* = 44), thyroid gland (*n* = 32) or gastric tumors (*n* = 18), review articles (*n* = 13), one study looked at the role of Tregs in periodontal disease and two articles were excluded because they assessed the expression of Foxp3 in tumor cells rather than assessing Tregs. Figure 1 shows the flow diagram of the studies retrieved for this systematic review.

### Data summary

The full characteristics of the study populations are displayed in Table 1.

**Table 1:** Basic information about the 45 studies that met the inclusion criteria for this systematic review.

**References**	**Tumor type**	**Tumor site**	**Number of samples**	**Sample**	**Species**
Stasikowska-Kanicka et al. (20)	SCC	Oral cavity (floor of the mouth)	78 Patients (41 poor prognosis, 37 better prognosis) 18 Controls (normal mucosa)	Tumor	Human
Hussaini et al. (31)	SCC	Oral cavity	25 Patients 12 Controls (inflammatory hyperplastic tissue)	Tumor	Human
Ihara et al. (17)	SCC	Oral cavity, oropharynx, nasopharynx, larynx, maxillary sinus	46 SCC 23 Controls	Blood	Human
Ma et al. (32)	SCC	HNC	**Human samples:** 43 Normal 48 Dysplastic 165 Primary HNSCC 12 Recurrent HNSCC 17 HNSCC with induction chemotherapy **Murine samples:** 6 WT normal tongue 6 WT tumor bearing mice 6 KO tumor bearing mice	Tumor	Human/Murine
Zhou et al. (33)	SCC	Tongue	46 SCC 46 Paired tumor adjacent non-neoplastic tongue epithelium 20 Metastasis lymph nodes 20 Paired normal cervical lymph nodes	Tumor	Human
Nguyen et al. (25)	SCC	Larynx, oral cavity, oropharynx, hypopharynx	278 SCC	Tumor	Human
Miki et al. (34)	SCC	Tongue	20 Controls 20 4NQO 40 4NQO treated with COX-2 inhibitor	Tumor	Murine
da Cunha Filho et al. (35)	SCC	Low lip	50 Patients, 10 microscopic fields per patient	Tumor	Human
Montler et al. (36)	SCC	Base of the tongue, tonsil, oropharynx, nasal, oral tongue, mandibular gingiva, maxillary sinus, larynx, floor of the mouth	29 Patients	Tumor blood	Human
Takahashi et al. (37)	SCC	Oral cavity, oropharynx, hypopharynx, larynx, paranasal cavity	20 Healthy controls 44 Patients treated with surgery/radio/radio-chemotherapy 16 Chemotherapy	Blood	Human
Jie et al. (18)	SCC	Oral cavity, oropharynx, larynx, hypopharynx	22 Patients treated with cetuximab plus cisplatin/paclitaxel/radiotherapy followed by 6 months of maintenance single agent cetuximab 18 Patients received single-agent cetuximab	Tumor blood	Human
Partlova et al. (38)	SCC	Tongue, tonsil, larynx, verbal base, hypopharynx, Gl. submandibularis, floor of mouth	54 Patients	Tumor blood	Human
Wolf et al. (29)	SCC	Oral cavity: tongue, upper alveolus, floor of mouth, hard palate, buccal mucosa, and retromolar	39 Patients	Blood	Human
Sun et al. (39)	SCC	Oral cavity, hypopharynx, nasopharynx, oropharynx, larynx	112 Patients 31 Healthy donors	Blood	Human
Schipmann et al. (40)	SCC	Oral cavity and skin	**FOXP3 mRNA expression:** 13 Cutaneous cSCC 8 Oral SCC 14 SCC metastases **Immunohistochemistry:** 10 Cutaneous SCC 8 Oral SCC 4 SCC metastases 10 Normal skin control **Cell lines:** Primary human adult skin fibroblasts Human squamous cell carcinoma cell line	Tumor	Human
Lim et al. (23)	SCC	Oral cavity	39 Patients 24 Controls	Blood	Human
Hanakawa et al. (16)	SCC	Tongue	34 Patients	Tumor	Human
Ward et al. (28)	SCC	Oropharynx	149 HPV+ 121 HPV–	Tumor	Human
Lukesova et al. (24)	SCC	Oral cavity, oropharynx	60 Patients	Blood	Human
Zhao et al. (41)	SCC	Tongue	16 Controls 32 4NQO	Tumor blood	Rat
Jie et al. (42)	SCC	Oral cavity, oropharynx	27 Patients	Tumor blood	Human
Park et al. (26)	SCC	Tonsil	79 Patients	Tumor	Human
Weed et al. (43)	SCC	Tongue	49 Patients	Tumor	Human
Drennan et al. (44)	SCC	Oropharynx, larynx	14 Controls 39 Patients	Blood	Human
Bron et al. (22)	SCC	Oral cavity, oropharynx, hypopharynx, larynx	35 Patients	Tumor blood	Human
Judd et al. (19)	SCC	Oral cancer cell line injected in flank	Not disclosed in manuscript	Tumor	Murine
Gaur et al. (45)	SCC	Oral cavity	45 Patients 40 Controls	Blood	Human
Wansom et al. (27)	SCC	Oropharynx	46 Patients	Tumor blood	Human
Wild et al. (46)	SCC	Oral cavity, pharynx, larynx	35 Patients 17 Controls	Tumor blood	Human
Näsman et al. (47)	SCC	Tonsil	31 HPV+ with a good clinical outcome 21 HPV+ with a poor clinical outcome 11 HPV– with a good clinical outcome 20 HPV– with a poor clinical outcome	Tumor	Human
Lee et al. (48)	SCC	Oral cavity	38 Patients 5 Controls	Tumor blood	Human
Schuler et al. (49)	SSC	Oral cavity, pharynx, larynx	9 Patient samples for dendritic cell culture 13 Patient samples for Treg frequency	Blood	Human
Alhamarneh et al. (50)	SCC	Larynx, oropharynx, oral cavity, hypopharynx, nasal cavity, lymph node metastasis, unknown primary site	107 Patients pretreatment 43 4–6 weeks posttreatment 40 Controls	Blood	Human
Al-Qahtani et al. (51)	SCC	Oral cavity	34 Patients	Tumor	Human
Tominaga et al. (52)	MM	Oral cavity	7 Patients 10 Controls	Tumor Blood	Canine
Horiuchi et al. (53)	MM	Oral cavity	15 Patients 10 Controls	Blood	Canine
Schott et al. (54)	SCC	Epipharynx, oropharynx, hypopharynx, larynx, oral cavity	16 Patients with active disease 16 Patients with no evidence of disease 21 Controls	Blood	Human
Gasparoto et al. (55)	SCC	Oral cavity, lip	9 Patients 10 Controls	Tumor Blood	Human
Boucek et al. (15)	SCC	Oral cavity, hypopharynx, larynx	112 Patients 20 Controls	Blood	Human
Distel et al. (56)	SCC	Oral cavity, hypopharynx, oropharynx	62 Low-risk group patients with early disease 53 High-risk group inoperable patients with advanced disease	Tumor	Human
Schwarz et al. (57)	SCC	Oral cavity	15 Patients 15 Controls	Tumor	Human
Bergmann et al. (58)	SCC	HNSCC cell lines from primary tumors	Cell culture of irradiated HNSCC cell lines from primary tumors with blood samples from 10 healthy donors	Blood	Human
Chikamatsu et al. (59)	SCC	Oral cavity, oropharynx, hypopharynx, larynx, paranasal sinuses	43 Patients 24 Controls	Blood	Human
Badoual et al. (21)	SCC	Oral cavity, oropharynx, hypopharynx	84 Patients	Tumor	Human
Schaefer et al. (60)	SCC	Larynx, oral cavity, pharynx, hypopharynx	24 Patients 17 Controls	Blood	Human

*SCC, Squamous Cell Carcinoma; MM, Multiple Myeloma; HNC, Head and Neck Cancer; HNSCC, Head and Neck Squamous Cell Carcinoma*.

#### Tumors

The majority of the studies [*n* = 39 (86.7%)] assessed human samples (15–18, 20–29, 31, 33, 36–40, 42–51, 54–60), one study assessed both human and murine samples (32), two studies looked at murine samples (19, 34), one at rat (41), and two at canine samples (52, 53). With the exception of two studies that looked into multiple myeloma in canines (52, 53), all studies focused on oral and/or oropharyngeal SCC [*n* = 43 (95.5%)]. Within the 45 studies, the site of the tumor varied and included tumors of the oral cavity (tongue, floor of the mouth, base of the tongue, gingiva), oropharynx, hypopharynx, lower lip, tonsil, epipharynx, and lymph node metastasis. Three studies used head and neck cancer cell lines (19, 40, 58).

Table 2 details the methodologies used to detect Tregs, the markers used and the changes in Tregs observed with treatment and with disease progression. Table 2 also summarizes the suggested role of Tregs in oral and oropharyngeal cancer for each included manuscript.

**Table 2:** Method and markers used to detect Tregs, and reported role of Tregs in HNC.

**References**	**Method for detecting Treg**	**Treg markers**	**Changes in Tregs observed with treatment**	**Changes in Tregs observed with disease progression**	**Suggested role of Tregs**
Stasikowska-Kanicka et al. (20)	IHC, morphometry	Foxp3+	N/A	The mean number of Foxp3+ cells was significantly increased in poor prognosis group in comparison to the better prognosis and control groups	Higher mean numbers of Tregs associated with poorer prognosis
Hussaini et al. (31)	IHC, IF	FoxP3+, TLR2	N/A	Significantly more single-stained FoxP3+ cells and double-stained FoxP3+TLR2+ cells in the OSCC than in the control group	FoxP3+TLR2+ cells may represent dendritic cell dependent pathway of inhibiting Treg suppression. Exact role in disease progression not disclosed
Ihara et al. (17)	FC	CD4, CCR4, CD127^low^, CD45RA-, Foxp3^high^	Low frequency of CD45RA–Foxp3 High Tregs before treatment showed a better clinical outcome, even in patients with advanced stage tumors		High frequency of CD45RA–Foxp3^high^ Tregs correlated with a poor prognosis and recurrence
Ma et al. (32)	FC, IHC, IF, WB	CD4, Foxp3, A2AR	A2AR blockade reduces CD4+ Foxp3+ Tregs in HNSCC mouse model. A2AR blockade enhances the anti-tumor response of CD8+ T cells in HNSCC mouse model	A2AR was correlated with higher pathological grade and significantly correlated with Foxp3	A2AR blockade reduces CD4^+^ Foxp3^+^ Tregs in HNSCC mouse model and enhances the anti-tumor response of CD8+ T cells
Zhou et al. (33)	IHC	CD4, FoxP3	N/A	Increased number of Tregs in SCC and metastatic lymph nodes Tissue in comparison to adjacent tissues	Expression of Tregs in SCC lesions was inversely associated with overall survival and associated with worse prognosis
Nguyen et al. (25)	IHC	FoxP3	N/A	N/A	Higher levels of FoxP3 infiltrates were associated with improved overall survival but not for relapse free or disease specific outcomes
Miki et al. (34)	Histopath, RT-PCR, IHC	Foxp3	No difference in the number of Foxp3+ cells between the control group and the groups treated with the COX-2 inhibitor regardless of the dose of COX-2 inhibitor	Foxp3 expression in the tongues of mice treated with 4NQO was significantly higher than normal control group (weeks 15 and 20), but significantly decreased with tumor progression	The authors could not conclude the exact role of Tregs in SCC
da Cunha Filho et al. (35)	Histopath, IHC	FoxP3+	N/A	Decrease in FoxP3+ T Cells with more advanced lesions and lymph node metastasis	Tregs are probably involved in early stages of lip carcinogenesis. Exact role not concluded
Montler et al. (36)	IHC, FC	CD25, Foxp3, OX40, PD-1, CTLA-4	N/A	N/A	High expression of OX40, as well as CTLA-4 and PD-1 in the TIL Tregs. Role in SCC not concluded
Takahashi et al. (37)	FC	CD3, CD4, CD25, CD127^low^	The proportion of Tregs decreased significantly at day 6 following treatment, but the activation marker increased at day 21	The proportion of Tregs was significantly higher in SCC patients compared to healthy donors	Chemotherapy can trigger a transient reduction of Tregs associated with an activation of CD8 T cells suggesting a tumor progressive role of Tregs in HNC
Jie et al. (18)	FC	CD4+, CD25^hi^,Foxp3+, CTLA-4, TGF-β, CD39	Cetuximab significantly increased the frequency of intratumoral Treg expressing CTLA-4, CD39, and TGF-β. significant increase was only observed in circulating Treg expressing CTLA-4		The frequency of CTLA-4+ Treg were significantly increased among the non-responder patients
Partlova et al. (38)	FC, RT-PCR	CD4+, CD25+, CD127^low^	N/A	N/A	No statistically significant differences were observed in the numbers and proportions of Tregs were observed between HPV+ and HPV– tumors. The role of Treg could not be concluded
Wolf et al. (29)	IHC	Foxp3	N/A	Levels of Tregs were higher in early stage cancers. Mean TIL levels for CD4, CD8, and FoxP3 cells were significantly correlated with each other and were higher in surviving patients	The findings suggest that Tregs are associated with better survival
Sun et al. (39)	FC	CD3, CD4, CD45RA–, Foxp3, CD25	N/A	Tregs increase in the peripheral circulation of HNSCC patients, and correlate with tumor stage and nodal status	The findings suggest a role for Treg in tumor progression
Schipmann et al. (40)	IHC, RT-PCR	Foxp3	N/A	Foxp3 expression much higher in SCC compared to normal controls	Oral and skin SCC recruit Tregs into the tumor microenvironment to suppress immunosurveillance
Lim et al. (23)	FC, ELISA	CD4+CD25^hi^CD127^low^	N/A	Tregs increased in SCC compared to normal controls	High levels of CD4+CD25^high^CD127^low^ Tregs is associated with better survival
Hanakawa et al. (16)	IHC	Foxp3	N/A	N/A	High intraepithelial and stromal infiltration of Tregs correlated with significantly worse 5-year disease-free survival
Ward et al. (28)	IHC	Foxp3	N/A	The proportion of Foxp3+ cells was reduced in HPV+ compared with HPV– tumors	Tregs were associated with improved survival, but might be a reflection of the overall increase in TIL
Lukesova et al. (24)	FC, PCR, IHC	CD4, CD3, CD25	N/A	Higher numbers of Tregs in oral tumors than oropharyngeal tumors	High level of Tregs in blood is associated with better survival
Zhao et al. (41)	FC	CD4+ CD25+ FoxP3+	N/A	Tregs were significantly higher in OSCC than controls and increased with the progression of 4NQO-induced rat tongue carcinogenesis	The results of this study suggest a role for Tregs in tumor progression
Jie et al. (42)	FC	CD4+, CD25+, Foxp3+ TGF-β CD39 CTLA-4	N/A	Intratumoral Treg exhibited more suppressive activity than peripheral blood Treg	The findings of this study suggest a suppressive function associated with disease progression
Park et al. (26)	IHC	Foxp3+ CD25+	N/A	Foxp3 expression is associated positively with p16 expression, and is a favorable prognostic factor for overall survival	Tregs are up-regulated in HPV+ SCC and Foxp3 is related to a favorable prognosis
Weed et al. (43)	IF	CD4+, Foxp3+	N/A	Cytoplasmic Foxp3 is associated with a lower possibility of recurrence, while nuclear Foxp3 is associated with a higher possibility of recurrence	The overall expression of FoxP3 does not Correlate with Clinical Outcome. However, elevated number of TILs expressing Foxp3 in the cytoplasm are indicative of a favorable prognosis while TILs expressing nuclear Foxp3 are associated with recurrence
Drennan et al. (44)	FC	CD4+ CD25^high^ CD127^low^	N/A	Level of peripheral Tregs increased with advanced tumor stage and lymph node involvement	The findings of this study suggest a role for Tregs in tumor progression
Bron et al. (22)	IHC	Foxp3	N/A	Treg more frequent in patients without lymph node involvement	High numbers of total FOXP3+ Tregs within the TIL were significantly associated with prolonged overall survival
Judd et al. (19)	FC	CD4+, Foxp3	Depletion of Tregs using anti-CD25 antibody resulted in a decrease in growth rate	N/A	Tregs contribute to the aggressive tumor growth in the studied model
Gaur et al. (45)	FC	CD4+ CD25+ Foxp3+	N/A	Increase in Th17/Tregs ratio in early stages and a decrease in this ratio in later stages due to a higher frequency of Tregs in later stages and in lymph node metastasis	The findings of this study suggest that Tregs are associated with more advanced disease and promote metastasis
Wansom et al. (27)	IHC	FOXP3	N/A	N/A	Higher levels of Tregs (Foxp3+) in TIL was associated with better disease specific and overall survival
Wild et al. (46)	FC, IF, RT-PCR, ELISA	CD4+, CD25+, FoxP3+, CD127^low^, TLR4	N/A	HMGB1 promotes suppressive function of Treg in HNSCC patients	The findings of this study suggest a role for Tregs in immune escape and tumor progression
Näsman et al. (47)	IHC	Foxp3	N/A	Higher number of Foxp3+ TILs HPV+ compared to HPV- SCC. No difference in Treg levels between poor and good prognosis	Although a High CD8+/Foxp3+ Ratio is Linked to a Good Clinical Outcome, no diff in Treg levels was observed related to clinical outcomes, indicating that the better prognosis is attributed to the elevated CD8 Levels
Lee et al. (48)	FC, IHC, RT-PCR	Foxp3, CD4, CD25, ICOS, TGF-β, CCR6	N/A	Within the TILs, the percentages of Th17 and Treg cells were inversely correlated. The prevalence of IL-17-producing FOXP3+ CD4+ in TIL is increased in SCC and possess suppressive function similar to Tregs	The findings of this study suggest a tumor promoting role for Tregs (regardless of their IL-17 production ability)
Schuler et al. (49)	FC	CD4+, CD25^high^, Foxp3+	RCT had diverse effects on Treg frequency	The mean frequency of Tregs was significantly increased SCC prior to Rct compared to healthy controls	Although this study reported unpredictable effect of RCT on Tregs, it reported an increase in Tregs in SCC patients suggesting an active mechanism of immune escape and tumor promotion
Alhamarneh et al. (50)	ELISA, FC	CD4+CD25^high^, GITR, CTLA-4, Foxp3	Post-treatment Treg levels were significantly higher than pre-treatment levels	Patients had significantly higher percentages of circulating Tregs compared with normal controls	The levels of Treg cells were elevated significantly SCC, however, they failed to correlate with disease progression or tumor burden
Al-Qahtani et al. (51)	IHC	Foxp3	N/A	Treg levels were higher in poorly differentiated SCC.	A linear positive correlation was established between tumor grade and number of Tregs suggesting a role in tumor promotion
Tominaga et al. (52)	IF, FC	CD4+, FoxP3+	N/A	Dogs with MM had increased numbers of circulating Tregs and TILs compared to healthy control dogs	The findings suggest a tumor promoting effect of Tregs
Horiuchi et al. (53)	FC	CD4+, Foxp3+	N/A	The percentage of circulating Treg increased with the tumor stage in dogs with oral MM	The findings of this study suggest Tregs possess a suppressive role for anti-tumor immunity, thus promoting tumor progression
Schott et al. (54)	FC	CD4+, CD25^high^, GITR, CTLA-4, CD122, CD127^low^, CCR7, Foxp3, CCL22	Increased Treg levels were found even in patients with no active disease several years after tumor resection	Increased ratio of Tregs within total CD4+ population in SCC patients. Increased level of GITR and CCR4 expression in Tregs from SCC patients	Increased Tregs in SCC patients might correspond to reduced anti-tumor immunity and therefore contribute to tumor progression or recurrence
Gasparoto et al. (55)	FC	CD4, CD25, FoxP3, GITR, CD45RO, CD69, TGF-β, CTLA-4, CCR4, IL-10	N/A	High frequency of Tregs in SCC patient blood with stronger suppressive ability than Tregs from healthy donors	Tregs suppress immune responses both systemically and in the tumor microenvironment, thus promoting tumor progression
Boucek et al. (15)	FC	CD3+, CD4+, CD25	N/A	Treg counts were higher in SCC patients compared to controls and were higher in recurrent disease	The levels of Treg in the peripheral blood correlate with a higher probability of early recurrence of SCC
Distel et al. (56)	IHC	Foxp3	N/A	In the low risk group, CD3+/Foxp3+ ratio had a clear impact on NED-survival with a low ratio being associated with a better prognosis. This was not observed in high risk patients	The results of this study suggest that intratumoral Treg infiltration on its own does not have an impact on tumor control or survival rates. CD3+/Foxp3+ ratio impacted NED-survival in the low risk group
Schwarz et al. (57)	IHC	Foxp3, CD25	N/A	Tregs were significantly elevated in SCC compared to control tissues	The authors could not conclude the role that Tregs play in tumor progression
Bergmann et al. (58)	FC, ELISA, WB	CD3, CD4,CD25+, Foxp3, IL-10, CTLA-4	N/A	overexpression of COX-2 and secretion of PGE2 by tumor cells induce the highly suppressive type 1 Treg (Tr1) subset of suppressor cells	The induction of Tr1 suppressor cells by SCC contribute to carcinogenesis by creating a suppressive microenvironment that promotes tumor growth
Chikamatsu et al. (59)	FC	CD4+, CD25+	N/A	Circulating Tregs are increased in patients with SCC compared to controls	Although there were no associations between Treg and tumor stage or histological differentiation, Treg percentage inversely correlated with that of total CD8+ T cells in cancer patients and was associated with inhibition of cytokine expression in CTLs suggesting a possible role in the downregulation of antitumor immune response
Badoual et al. (21)	IF	CD3, CD4, CD25, Foxp3, CD69	N/A	Overall, high levels of CD4+CD69+, CD4+CD25+ or CD4+Foxp3+ are associated with better survival and locoregional control	The findings of this study suggest that tumor infiltrating Tregs are associated with a better prognosis
Schaefer et al. (60)	FC	CD3, CD4+, CD25+, Foxp3, GITR, CCR7	N/A	Patients had significantly higher percentages of circulating Tregs than controls	Although the effect of Treg on downregulating the immune functions of other T cells subsets was shown, the exact role of Treg on disease progression could not be confirmed in this study

*FC, Flow Cytometry; IHC, Immunohistochemistry; IF, Immunofluorescence; WB, Western Blot*.

#### Samples and treg analyses

Eighteen studies assessed tumor samples (16, 19–21, 25, 26, 28, 31–35, 40, 43, 47, 51, 56, 57), 16 assessed blood samples (15, 17, 23, 24, 29, 37, 39, 44, 45, 49, 50, 53, 54, 58–60), and 11 studies assessed both tumor and blood samples (18, 22, 27, 36, 38, 41, 42, 46, 48, 52, 55).

With regards to the methodologies used to detect and assess Tregs, immunohistochemistry was used in 21 studies (16, 20, 22, 24–29, 31–36, 40, 47, 48, 51, 56, 57), flow cytometry in 25 studies (15, 17–19, 23, 24, 32, 36–39, 41, 42, 44–46, 48–50, 52–55, 58–60), Immunofluorescence in six studies (21, 31, 32, 43, 46, 52), PCR in six studies (24, 34, 38, 40, 46, 48), ELISA in four studies (23, 46, 50, 58), Histopathology and morphology in three studies (20, 34, 35), and Western blots in two studies (58, 32). The majority of the studies (30 out of 45 studies) used only a single method for detecting Tregs (15–19, 21, 22, 25–29, 33, 37, 39, 41–45, 47, 49, 51, 53–57, 59, 60). The most common single method was flow cytometry (16 out of 30) (15, 17–19, 37, 39, 41, 42, 44, 45, 49, 53–55, 59, 60), followed by immunohistochemistry (12 out of 30) (16, 22, 25–29, 33, 47, 51, 56, 57) and immunofluorescence was used as a single method for Treg detection in two studies (21, 43). Eight out of the 45 studies used two methods of Treg assessment (20, 23, 31, 35, 36, 40, 50, 52) while seven studies used three or more methodologies (24, 32, 34, 38, 46, 48, 58).

As for the markers used to detect Tregs and assess their function, Foxp3 was the most commonly used marker, as it was used in 38 out of the 45 studies (16–22, 25–29, 31–36, 39–43, 45–58, 60). Foxp3 was the sole marker for Treg detection in 13 studies (16, 20, 22, 25, 27–29, 34, 35, 40, 47, 51, 56). Foxp3 in combination with T cell markers CD3, CD4, and/or CD25, was used as a marker in 14 studies (15, 19, 24, 26, 33, 41, 43, 45, 49, 52, 53, 57, 59). CD25 was used as a marker (on its own or with other markers) in 24 studies (15, 18, 21, 23, 24, 26, 36–39, 41, 42, 44–46, 48–50, 54, 55, 58–60). Seven studies identified Tregs using a combination of CD4+CD25+CD127^low^ with or without other markers (17, 23, 37, 38, 44, 46, 54). CTLA-4 was used as a marker of Treg phenotype or suppressive function in seven studies (18, 36, 42, 50, 54, 55, 58), GITR was assessed in four studies and as a marker of Treg function (50, 54, 55, 60), TGF-β was assessed in four studies (18, 42, 48, 55), and IL-10 was used in two studies (55, 58).

#### HPV status

Only 11 manuscripts looked at the HPV status of the tumors (24–29, 32, 36, 38, 43, 47), and out of those, only 10 included HPV positive cases in their studies (24–29, 32, 36, 38, 47). Half these studies reported no difference in Treg levels between HPV positive and negative tumors (24, 27, 29, 32, 36). Two manuscripts reported a decrease in Treg proportion in HPV positive (28, 38) [one associated with an increase in TIL (28)], and three reported an increase in Treg associated with an overall increase in TIL (24, 25, 47). Four studies associated HPV positive tumors with better survival compared to HPV negative (24, 27, 28, 47). One study found no correlation between HPV status and survival (29). Three studies correlated an increase in Tregs in HPV positive tumors with better prognosis (24, 26, 28), however, one of the studies suggested that it was associated with the overall increase in TIL (28).

#### Correlation of tregs with clinical outcome

Twenty-four studies reported a clear increase in Tregs (whether intratumoral or circulating) in cancer patients in comparison to healthy controls and/or in more advanced disease (15, 20, 23, 31–34, 37, 39–41, 44, 45, 48–55, 57, 59, 60). Only three studies reported a decrease in Tregs with more advanced disease (22, 29, 35).

Out of the 45 papers included in this study, 25 studies (55.6%) found a correlation between Tregs and poor clinical outcome and disease progression (15–20, 32, 33, 37, 39–42, 44–46, 48, 49, 51–55, 58, 59), nine manuscripts (20%) correlated Tregs to good clinical outcome (21, 22, 23, 24, 25, 26, 27, 28, 29), and 11 (24.4%) did not reach a conclusion regarding the role of Tregs in tumor progression (31, 34–36, 38, 43, 47, 50, 56, 57, 60).

No apparent correlation was found between the site of the tumor and the outcome. Only one study reported higher numbers of Tregs in Oral SCC lesions in comparison to oropharyngeal tumors (24).

With regards to the type of samples assessed for Tregs, interestingly, the majority of the studies that could not conclude the role of Tregs [7 out of 11 studies (63.6%)] looked only at tumor samples (31, 34, 35, 43, 47, 56, 57). Four of the studies that only assessed tumor samples showed an association between Tregs and good prognosis (21, 25, 26, 28), while seven showed association with poor prognosis and clinical progression (16, 19, 20, 32, 33, 40, 51). Regarding the studies that assessed blood samples only, the majority [11 out of 16 (68.7%)] reported an association between Tregs and poor outcome (15, 17, 37, 39, 44, 45, 49, 53, 54, 58, 59), while three studies reported a good outcome (23, 24, 29) and two studies could not conclude a definite role (50, 60). When both tumor and blood samples were assessed, only two studies (out of 11) could not define a role for Tregs in disease progression (36, 38), two studies found a positive correlation with good outcome (22, 27) while the remaining seven studies reported a correlation with poor outcome and disease progression (18, 55, 42, 48, 46, 41, 52).

Regarding the method used to detect Tregs, interestingly, flow cytometry was only used in two of the nine studies that concluded a good prognosis (23, 24), and four out of the studies that made no conclusion (50, 36, 38, 60). On the other hand, immunohistochemistry was used in seven out of the nine studies that found a positive connection between Treg and a better clinical outcome (22, 24, 25, 26, 27, 28, 29), and seven out of the studies that made no conclusion (31, 34–36, 47, 56, 57).

As for the markers used, remarkably, Foxp3 was the only marker used to identify Tregs in five out of the nine studies that suggested a positive clinical outcome with an increase of Tregs (22, 25, 27, 28, 29). Five out of the seven studies that used CTLA-4 as a marker of Treg correlated the presence of Treg with a poor clinical outcome (18, 58, 55, 42, 54). The remaining two did not conclude a definitive role for Tregs (50, 36). On the other hand, out of the four studies that assessed GITR, two associated Tregs with poor clinical outcome and disease progression (55, 54), while the other two did not conclude a role (50, 60). All four studies that assessed TGF-β as a marker of Treg function found a negative clinical outcome associated with Treg (18, 55, 42, 48).

## Discussion

Cancer immunotherapy to reactivate anti-tumor immunity is one of the most important recent developments in cancer treatment. For some patients, targeting the immune system to boost its anti-tumor activity can generate enduring disease remission, but despite the impressive successes in cancer immunotherapy, the response in patients is sometimes transient. This is attributed to multiple factors including the exhaustion of tumor-specific CD8 T cells in addition to induced suppression of the immune response against cancer. One of the major immune escape mechanisms in cancer patients is the conversion and dominance of suppressive immune cells within the tumor microenvironment that hamper the function of anti-tumor effector T cells. Regulatory CD4 T cells (Tregs) are among the most studied suppressor cells in the tumor microenvironment and their role in mediating tumor progression has been reported in many types of cancer. Indeed, reducing the number of Tregs has been reported to enhance anti-tumor immunity and promote tumor regression (9–14).

However, in head and neck cancer and particularly in OPSCC, the role of Tregs in mediating tumor progression and affecting the overall clinical outcome is not clear. In fact, there are conflicting reports in the literature; while a considerable number of studies reported a similar role for Tregs in mediating tumor escape mechanisms and facilitating tumor progression (15–20), other studies reported an opposite role and associated Tregs with a positive clinical outcome (21–29).

In this systematic review, we attempted to assess the body of knowledge available about the role that Tregs play in head and neck cancer with the aim of understanding the reasons for this contradiction in describing the role that Tregs play in disease progression and the clinical outcome.

Our findings emphasized the controversy in the literature. An elevated level of Tregs in patients was observed in some studies (15, 20, 23, 31–34, 37, 39–41, 44, 45, 48–55, 57, 59, 60), while no significant differences were reported between patients and healthy controls in others and a decrease in Tregs with more advanced disease was observed in three studies (22, 29, 35). While more than half of the reviewed studies reported a poor prognosis associated with increased levels of Tregs (15–20, 32, 33, 37, 39–42, 44–46, 48, 49, 51–55, 58, 59), many studies reported a better prognosis (21–29). A considerable number of studies did not conclude a role for Tregs in tumor progression or clinical outcome (31, 34–36, 38, 43, 47, 50, 56, 57, 60).

One of the potential reasons for the controversy in the literature, is different reports from different species. We therefore included all the manuscripts from all species to assess whether the species under study affected the reported outcome. The only study that assessed both human and murine samples reported a role for Tregs in promoting tumor progression (32). One of the two studies that assessed murine sample did not reach a conclusion about the role of Tregs (34), while the second murine study reported a role for Tregs in enhancing tumor progression (19). Similar results about the role of Tregs in promoting disease progression were reported in the only study that assessed rat samples (41) and the two canine samples (53, 52). These findings ruled out any role for inter-species variability in the controversy in the literature.

In recent years, the incidence of HPV positive oropharyngeal cancers has increased and is on the rise. Surprisingly, we report that HPV status was assessed in only 11 studies out of the 45 included in this systematic review. HPV-associated tumors are a distinct subtype with different intra-tumoral immune cell infiltration and better prognosis (24, 27, 28, 47). Therefore, phenotyping tumors according to their HPV positivity is essential when assessing the role of different immune cells in anti-tumor immunity.

Despite the advances in head and neck cancer diagnosis and treatment, the mortality rate is still high. This is mainly attributed to late diagnosis and the lack of predictors of disease progression. Premalignant lesions are altered tissues that carry a higher risk of developing into malignancy, but unfortunately markers to predict malignant transformation into malignancy in these lesions are lacking. Surprisingly, among all the reviewed articles in this study, only three studies assessed premalignant lesions in animal models (32, 34, 41), and only one of these studies assessed samples from human patients (32). All three studies reported an increase in suppressive Tregs with disease progression from normal through dysplastic to neoplastic lesions (32, 34, 41). In their study, Ma et al. reported a correlation between disease stage and Tregs and in particular in A2AR expression. They reported that blocking A2AR reduced Tregs in the tumor bearing mice and enhanced anti-tumor immune response (32). Understanding the immune response within premalignant lesions is crucial to predict their progression into malignancy and to design treatments to modulate the immune response to eliminate these lesions before transforming into cancerous lesions.

Interestingly, distinction between thymic vs. peripheral Treg within the tumor microenvironment was not made nor assessed in any of the identified manuscripts in this systematic review. This is not a surprise given the lack of markers that accurately determine the origin of Tregs, but certainly measuring intratumoral numbers of converted Treg, defined by markers such as CD103 or S1PR may yield more precision for the role that Treg play within the tumor microenvironment.

In this systematic review, we found that Foxp3 is the most commonly used marker for Treg identification. In fact, it was the only marker used in 13 studies (16, 20, 22, 25, 27–29, 34, 35, 40, 47, 51, 56). Many of the studies used immunohistochemistry and a single stain to detect Foxp3. This causes a potential problem as Foxp3 is highly expressed in other activated T cell subsets including effector T cells. In fact, it has been proposed that under certain inflammatory condition, Foxp3+ Tregs might become unstable adopting a phenotype that is more characteristic of effector CD4+ T cells (61). Foxp3 might be a marker of activation rather than a marker of regulation and a key identifier of Tregs. Therefore, using dual staining and co-localization of markers such as CD4 and Foxp3 could clarify the role of Treg TIL subset in the tumor microenvironment more accurately.

Furthermore, Foxp3 is expressed in tumor cells. In fact, it has been reported that tongue SCC tumor cells express Foxp3 and its expression significantly associated with disease progression and poor patient outcome (62). The same group found that Foxp3 expressed in tumor cells has distinct biological functions compared with that in Tregs (63). On the other hand, the expression of Foxp3 in tumor cells is associated with an increase in the secretion of sCTLA-4, which was recently reported to be a favorable predictor of clinical outcome in advanced cancers (64). This could explain the reported association between Tregs, as identified by Foxp3 expression, and a favorable clinical outcome.

This adds to the controversy regarding the role of Tregs and emphasizes the need to use more than one method and different markers to detect Tregs within the tumor microenvironment. Remarkably, immunohistochemistry was the only used method to assess Tregs in 21 studies (16, 20, 22, 25, 27–29, 34, 35, 40, 47, 51, 56). A study further suggests that the overall expression of Foxp3 in Tregs by itself is not an important predictor of clinical outcome, but rather the localization of Foxp3 is the important predictor of outcome. Weed et al. reported that in oral SCC, nuclear Foxp3 is associated with a higher probability of early disease recurrence in comparison to cytoplasmic Foxp3, which is associated with a lower probability of recurrence (43). Immune profiling and the pattern of TIL within the tumors is of great importance as reported by Feng et al. after we conducted our search. They reported that the distance between the suppressive Foxp3+ Tregs and the effector CD8+ T cells are predictive of patient overall survival (65). Surprisingly, despite the great advances in different assays that added to the confidence in defining Treg markers, our study did not find any major changes in the assays or markers used to detect Tregs over the 12 year period (2005-2017) that the manuscripts included in this review covered. These reports emphasize the importance of using different markers, assays and analyses in the study of immune cells in cancer patients.

Checkpoint inhibitor antibodies represent a novel type of cancer immunotherapy that has seen notable success in the treatment of different cancers (66). One of the major targets of checkpoint inhibitors is CTLA-4, which is highly expressed on Tregs. Only seven studies out of the 45 studies that we assessed looked at the expression of CTLA-4 in Treg or used it as an identifier for these suppressor cells (18, 36, 42, 50, 54, 55, 58), five of which correlated the presence of Treg with a poor clinical outcome (18, 42, 54, 55, 58). One of these studies reported a higher frequency of CTLA-4+ Tregs (identified as CD4+CD25^hi^Foxp3+) in non-responder patients to Cetuximab (18), suggesting a potential use of this immune checkpoint as a biomarker for response to therapy.

GITR, another immune checkpoint was assessed in four studies as a marker of Treg function (50, 55, 60, 54), two of which associated Tregs with poor clinical outcome and disease progression (55, 54). TGF-β was assessed in four studies as by the membrane bound form (LAP), or using RT-PCR (18, 55, 42, 48), all of which correlated Tregs to poor clinical outcome.

## Conclusions

In conclusion, our systematic review emphasized the existing controversy regarding the role of Tregs in head and neck cancer, and in particular in OPSCC. We conclude that similar to most cancer types, Tregs contribute to tumor escape mechanisms and are therefore associated with poor clinical outcome. The inconsistent results reported in the literature could be due to the use of different markers to identify Tregs, variation in patient recruitment criteria or a heterogeneous cancer population. Indeed, we observed major differences in the reported outcomes between studies that assessed tumor samples, and those that assessed blood samples, suggesting the need to assess both to reach a more definitive understanding of the role of different immune cells in disease progression. HPV status, an important prognostic marker in OPSCC, was not assessed in majority of the studies, which could explain some of the discrepancy in the findings.

Our findings therefore suggest the need to define a better and more robust method to detect Tregs in the tumor microenvironment and in the periphery using a combination of methodologies, markers and analyses. We suggest using a combination of markers to define Tregs in the periphery and within TIL, including CD4, CD3, CD25, CD127lo, FoxP3, and CTLA-4. We also propose incorporating the regulatory properties of tumor cells as well as TIL for a complete picture of the tumor microenvironment.

## Author contributions

CO performed the search, screening and data extraction. FW contributed to results interpretation and writing the manuscript. RA designed the study, checked the accuracy of the data extraction, and contributed to results interpretation and writing the manuscript.

### Conflict of interest statement

The authors declare that the research was conducted in the absence of any commercial or financial relationships that could be construed as a potential conflict of interest.
